# Phenotype from *SAMD9* Mutation at 7p21.2 Appears Attenuated by Novel Compound Heterozygous Variants at *RUNX2* and *SALL1*

**DOI:** 10.1055/s-0041-1740018

**Published:** 2022-06-13

**Authors:** E. Scott Sills, Samuel H. Wood

**Affiliations:** 1 Reproductive Research Section, Center for Advanced Genetics, San Clemente, California, United States; 2 Department of Obstetrics & Gynecology, Palomar Medical Center, Escondido, California, United States; 3 Gen 5 Fertility Center, San Diego, California, United States

**Keywords:** SAMD9, RUNX2, SALL1, phenotype, renal structure

## Abstract

Sterile α motif domain-containing protein 9 (SAMD9) is a regulatory protein centrally involved in cell proliferation and apoptosis. Mapped to 7p21.2, variants in *SAMD9* have been reported in <50 pediatric cases worldwide, typically with early lethality. Germline gain-of-function *SAMD9* variants are associated with MIRAGE syndrome (myelodysplasia, infection, restricted growth, adrenal hypoplasia, genital anomalies, and enteropathy). Spalt like transcription factor 1 (SALL1) is a zinc finger transcriptional repressor located at 16q12.1 where only two transcript variants in *SALL1* are known. *RUNX2* (6p21.1) encodes a nuclear protein with a Runt DNA-binding domain critical for osteoblastic differentiation, skeletal morphogenesis, and serves as a scaffold for nucleic acids and regulatory factors involved in skeletal gene expression. RUNX2 and SALL1 are thus both “master regulators” of tissue organization and embryo development. Here, we describe exome sequencing and copy number variants in two previously unknown mutations—R824Q in SAMD9, and Q253H in SALL1. A multiexon 3′ terminal duplication of *RUNX2* not previously encountered is also reported. This is the first known phenotype assessment for an intersection of all three variants in a healthy 46,XX adult. Focusing on developmental progress, ultrastructural renal anatomy, and selected reproductive aspects, we describe this unique genotype diagnosed incidentally during coronavirus disease 2019 (COVID-19) illness. Individually, disruption in *SAMD9, RUNX2,* or *SALL1* would be expected to give a bleak prognosis. However, this variant convergence appears to dampen severe pathology perhaps by cross-gene silencing of effects normally deleterious when such changes occur alone.

## Introduction

Mutations in SAMD9 (sterile α motif domain-containing protein 9) are usually recognized early in life, as adaptive immune response impairment leads to chronic childhood infections and high mortality by age 10. 1 2 *SAMD9* maps to chromosome 7q21.2, a region frequently deleted in myeloid malignancies. Narumi et al 3 were the first to implicate germline missense *SAMD9* mutations as causative for a rare condition known as MIRAGE syndrome (i.e., myelodysplasia, infection, growth restriction, adrenal hypoplasia, genital anomaly, and enteropathy). Such mutations were later identified in ∼18% of inherited bone marrow failure and myelodysplasia cases. 4 Regarding *RUNX2* , ∼200 different mutations are known and most are seen with cleidocranial dysplasia, an autosomal dominant skeletal disorder including clavicular dysmorphia, increased head circumference, large fontanels, dental anomalies, short stature, and sometimes hand malformations. 5 Spalt like transcription factor 1 gene ( *SALL1* ) modulates onset and progression of human tumors, with variants now known to be associated with Townes-Brocks syndrome (i.e., anal, renal, limb, and ear anomalies). 6 7 While disruptions in *SAMD9, RUNX2,* and *SALL1* have each been encountered separately, this report is the first to present data on simultaneous variants in all three.

## Clinical Presentation

This 18-year-old nonsmoking Caucasian female patient sought a second opinion consult regarding irregular menses. Academic progress was normal through high-school, with extracurricular activities including team sports and music. Accompanied by her mother, the patient appeared developmentally normal. Review of the prenatal chart was notable for small for gestational age diagnosis in the third trimester. A genetic amniocentesis was normal. She was delivered by cesarean at 34 weeks' gestation with birth weight 1,700 g. Placenta and umbilical cord were grossly normal. Bilateral choanal atresia was diagnosed while in neonatal nursery, and was successfully repaired by age 3 months.

Recent medical history was unremarkable, although mild intermittent macrocytic anemia was occasionally noted on complete blood count and required no treatment. There was no history of electrolyte imbalance. Blood type was O + . Menarche was at age 11, and normal ovarian, uterine, and cervical anatomy was noted on a prior pelvic computed tomography. Six months before assessment, the patient was evaluated elsewhere for persistent headache, low grade fever, diarrhea, emesis, and fatigue. Pregnancy test was negative. Coronavirus disease 2019 (COVID-19) screen was initially negative, but oral vancomycin was started for *Clostridium difficile* which was found incidentally. Symptoms failed to resolve, and temperature increased to 39.7°C within 2 days when repeat COVID-19 testing was positive. By now underweight, the patient was admitted to hospital where supportive care was provided in an isolation unit; oral vancomycin was adjusted to intravenous administration. Three days later, her body mass index was 16.8 and fever, diarrhea, and vomiting persisted. Proteinuria was noted with high serum creatinine (3.5 mg/dL). The diagnosis was revised to COVID-19-associated multisystem inflammatory syndrome in children and the patient was transferred to intensive care unit (ICU). Next, vancomycin was discontinued and remdesivir, azithromycin, dexamethasone, and intravenous immunoglobulin therapy began. 8 While in ICU, the patient's erythrocyte sedimentation rate, C-reactive protein, and D-dimer level (all of which had been markedly elevated) began to decline. After a 15-day hospitalization, she was discharged home with a recommendation for outpatient renal biopsy. This was completed along with a comprehensive genetics panel. Her gastrointestinal symptoms resolved as activities returned to normal, yet appetite remained low (thought to be COVID-19 sequela).

**Fig. 1:**
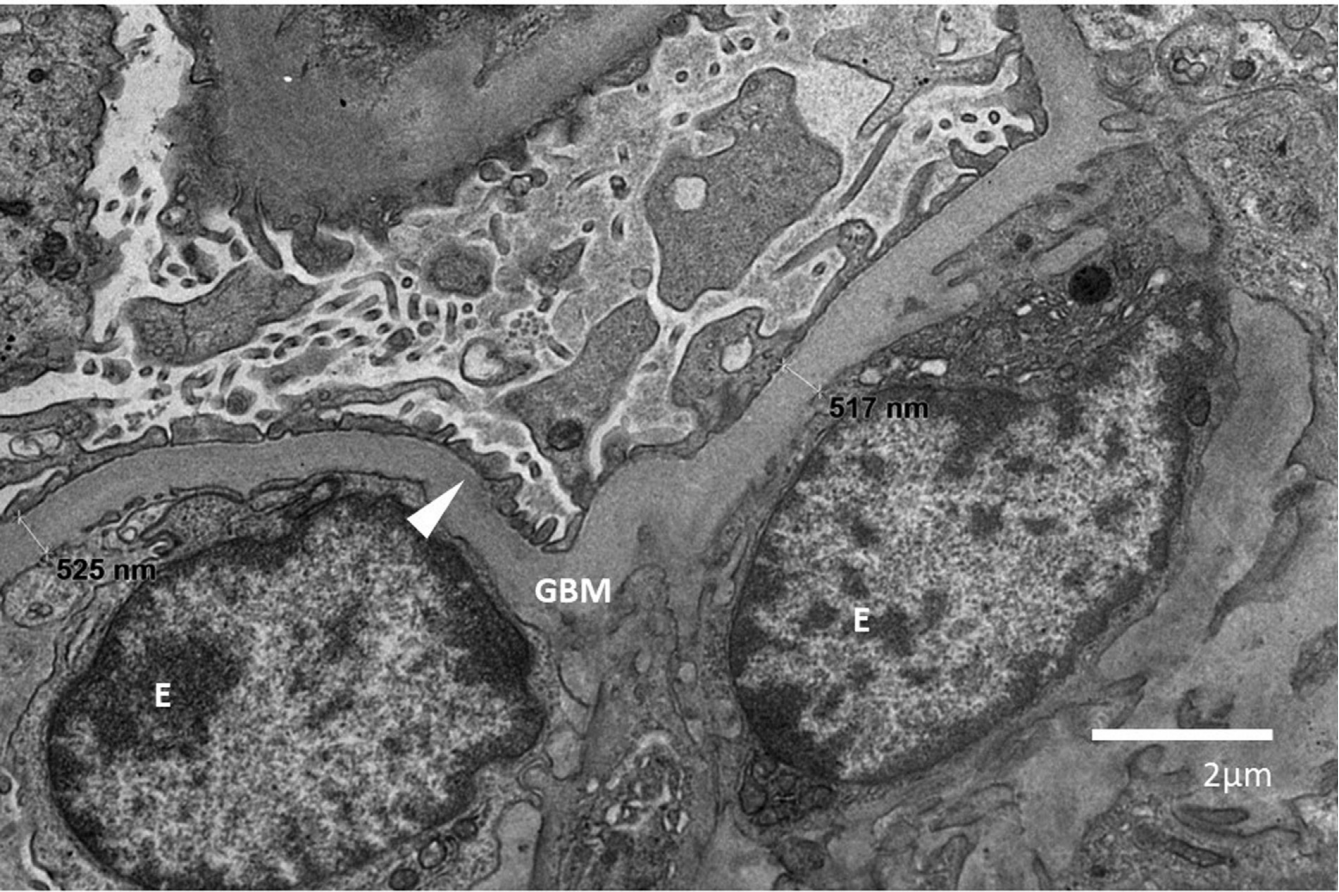
Transmission EM (1500x) view of renal tissue in a healthy 46,XX patient (age 17.5 years) with variants at *SAMD9* , *RUNX2* , and *SALL1* . Moderate focal global glomerulosclerosis is noted with segmental, irregular podocyte foot process effacement (arrow). Minimal basement membrane thickening is also present with no significant endothelial edema. Immunofluorescence stains were negative for IgA, IgM, C1q, C3, kappa, lambda, fibrinogen or properdin. E, endothelial cell; GBM, glomerular basement membrane.

**Fig. 2:**
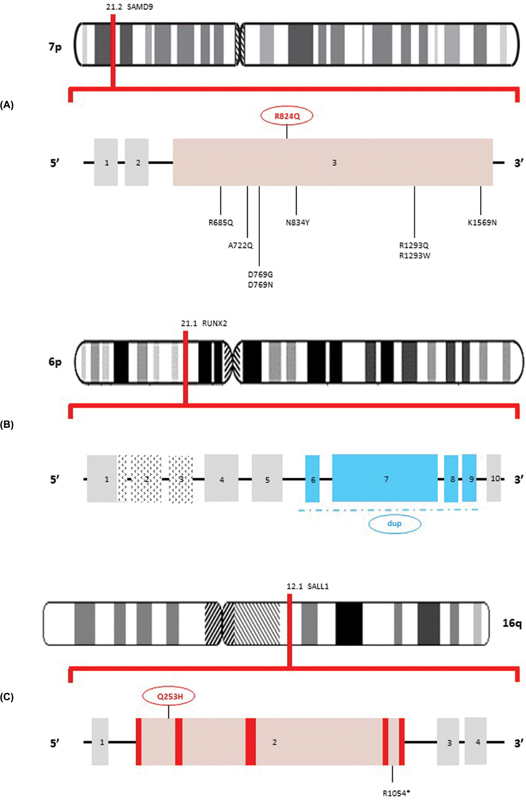
Schema for ( **A** ) variant at 7p21.2 *SAMD9* (R824Q, red oval) NM_017654.3. Also shown are ( **B** ) multiexon *RUNX2* duplication (blue oval) at 6p21.1 (NM_001024630.3) and ( **C** ) variant at 16q12.1 *SALL1* (Q253H, red oval) NM_002968.2. Of note, the newly discovered *RUNX2* and *SALL1* variants were also present in proband's mother.

Renal biopsy showed focal global and partial glomerulosclerosis, extensive foot process effacement, as well as mild interstitial chronic inflammation ( Fig. 1 ). These changes were regarded as consistent with recent COVID-19 infection. Exome sequencing identified three previously unknown variants ( Fig. 2 ). Protein variation effect analysis 9 was performed to estimate impact of amino acid substitution on resultant protein bioactivity by delta alignment score. 10 The new *SAMD9* variant was associated with a neutral effect (−0.5), although at *SALL1* the variant was considered deleterious (−4.08). Data were insufficient to develop an estimate for the observed *RUNX2* duplication (see Table 1 ). Analysis of mtDNA was normal.

**Table 1:** Summary of variant, protein, and genomic loci data present in 46,XX nonsyndromic SAMD9

	DNA coding	Protein product	Coordinates	var.effect a	Parental data
SAMD9	2471 G > A	Arg824Gln R824Q	7:92732940	−0.5	*de novo*
RUNX2 b	[ *dup* ]				mat.dup.
SALL1	759 A > T	Gln253His Q253H	16:51175374	−4.08	mat.var.

Note: Variants reported are inherited as heterozygous/autosomal dominant. [ *dup* ] = gene duplication.

a Calculated protein variation effect analysis (PROVEAN).

b 3' end duplication corresponds to arr[GRCh37] 6p21.1 (45459496_45515207)x3; same *RUNX2* variant also identified in mother.

## Genomic Methodology

Genomic DNA analysis was performed on cells sampled from proband and both parents by buccal swab technique. 11 Exomes and flanking splice junctions were captured using the IDT xGen Exome Research Panel v1.0 (Integrated DNA Technologies, Coralville, Iowa, United States). Massively parallel NextGen sequencing was done on an Illumina platform with ≥100bp paired-end reads. These were aligned to human genome build GRCh37/UCSC hg19, and analyzed for sequence variants using a custom-developed analysis tool. 12 Additional sequencing and variant interpretation were applied as described previously. 13 Variant classification criteria are available at GeneDx ClinVar page ( http://www.ncbi.nlm.nih.gov/clinvar/submitters/26957/ ).

## Discussion

Occurrence of *SAMD9* , *RUNX2* , and *SALL1* mutation in humans is rare and information on these events mainly comes from small series or case reports. Such data are valuable in providing improved knowledge of gene function, particularly when variants are linked to specific phenotypes. Because SAMD9 is an important regulatory protein crucial for normal development, it is unsurprising that variants in the responsible gene would yield an ‘orphan disease’ diagnoses in children and an unfavorable prognosis. 14 Yet the full significance of any discovered variant is best appreciated by consideration of the entire genome. The convergence we report here is believed to be the first instance where unique variants in *SAMD9* , *RUNX2* , and *SALL1* , all appear together. Accordingly, the phenotype assessment adds to the understanding of these loci and suggests how they may integrate to affect overall function.

For our patient, the provisional diagnosis of MIRAGE syndrome seemed a poor fit given her overall clinical picture and developmental history. While the association between *SAMD9* and MIRAGE syndrome is established, 3 in our case a theory was required to account for multiple missing elements. Prior reports on MIRAGE syndrome emphasized germline mutations, which were not proven here. Other possibilities included a reversion mutation, 15 or perhaps this variant in *SAMD9* was differentially operant on growth networks in multiple cell contexts. The latter option would align with how alterations in the Ras/mitogen-activated protein kinase (Ras/MAPK) pathway are considered to result in a range of pathologic conditions with distinct clinical phenotypes. 16 17 Cross-regulation by variants elsewhere could not be dismissed, however. Attention was thus turned to *RUNX2* and *SALL1* .

Spalt-like transcription factors (SALLs) are highly conserved proteins that direct embryo development, apoptosis, angiogenesis, invasion, and metastasis. 18 SALL1 is a transcription factor that mediates organogenesis and cell differentiation. 19 Since gene expression is slowed when DNA is loosely packed, and SALL1 can dampen this action by influencing how tightly DNA is packed, the presence of this new *SALL1* variant gained additional relevance. Moreover, an instance where SALL1 influences gene operation is already known: its role as a tumor suppressor in breast cancer, which is downregulated in estrogen receptor, progesterone receptor, and epidermal growth factor receptor-2 “triple negative” breast cancer patients. 20

Runt-related transcription factor 2 (RUNX2) regulates osteogenesis and shares common signaling pathways in other tissue development. It is known to cooccur in several epithelial and mesenchymal cancers, linked by multiple cancer-related proteins and microRNAs. 21 Crucially, experts in China discovered a role for *SALL1* in differentiation of murine odontoblast lineages specifically with *RUNX2* . 22 Our data support the observation of a coordinated regulatory role involving both *RUNX2* and *SALL1* , offering further justification for why all three genes should be considered together.

Several issues remain open and thus limit this report. For example, although examination of renal ultrastructure revealed nonspecific inflammatory changes, this may have been related to COVID-19 rather than any gene variant identified here (i.e., comparison versus preinfection tissue sample was not possible). Imaging tests showing grossly normal pelvic anatomy cannot forecast reproductive capacity into adulthood. And since our assessment was confined to exon sequencing only, any noncoding DNA mutation would be missed by this technique. 23 24 Nevertheless, the identification of these unexpected variants in *SAMD9, RUNX2* , and *SALL1* in a clinical setting of normal development offers new insights into how these loci may operate in concert.

In summary, the pathogenic effects of the de novo variant in SAMD9 appear attenuated by changes in RUNX2 and SALL1 operating concurrently. Careful longitudinal follow-up is warranted and data from further assessments will be reported when available.
